# Supplementary figures and images for: The bilevel chamber revealed differential involvement of vasopressin and oxytocin receptors in female mouse sexual behavior
Source: PLoS One. 2024 Jun 20;19(6):e0304703. doi: 10.1371/journal.pone.0304703 (PMC11189176; doi:10.1371/journal.pone.0304703)

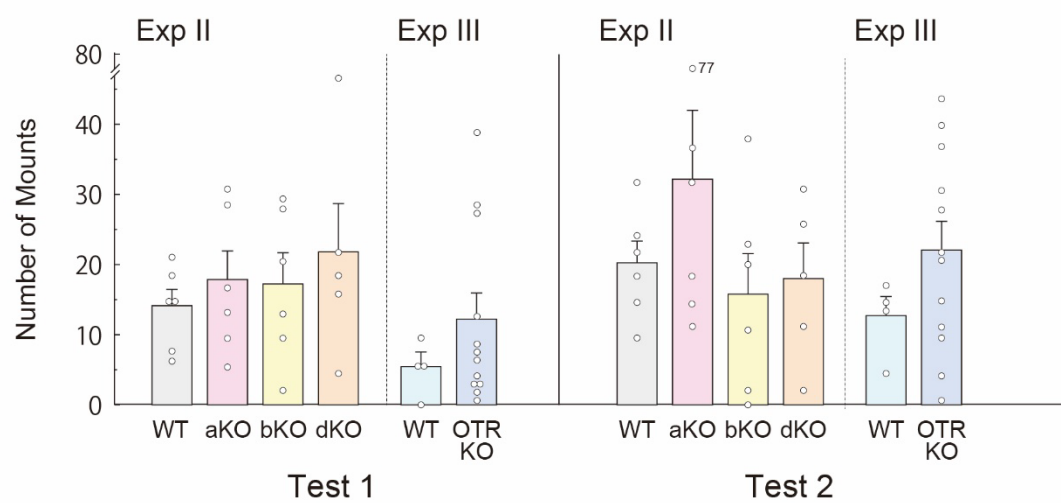

Supplement: S1 Fig — No significant difference was observed between any two genotype groups or tests. (PDF) [file pone.0304703.s001.pdf]

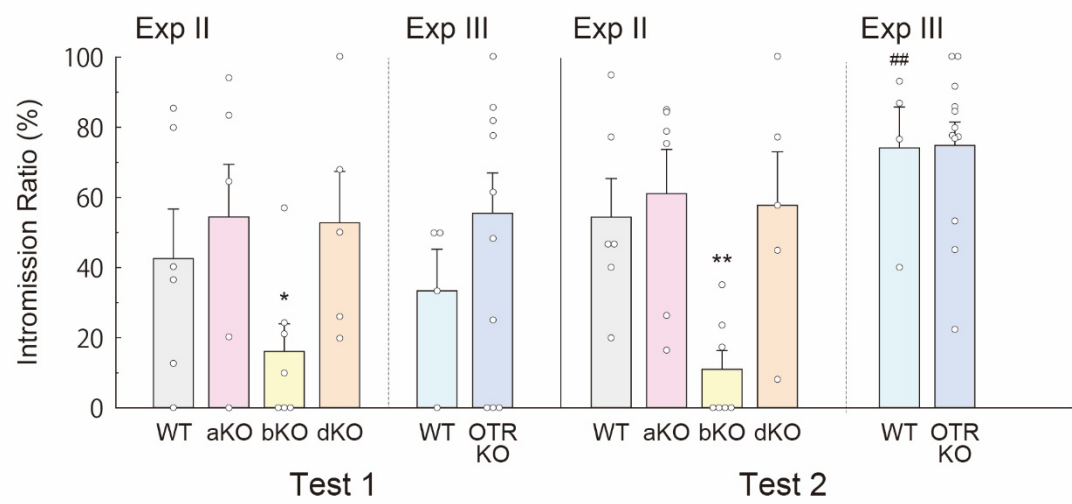

Supplement: S2 Fig — Results were statistically analyzed by ANOVA, followed by Bonferroni, *p < 0.05: significant difference between bKO and dKO (F1,40 = 3.05), **p < 0.01: significant difference between WT and bKO (F1,40 = 3.80)/bKO and dKO (F1,40 = 3.88), and ##p < 0.01: significant difference between Tests 1 and 2 (F1,14 = 4.19). (PDF) [file pone.0304703.s002.pdf]
