# Supplementary material for: The bilevel chamber revealed differential involvement of vasopressin and oxytocin receptors in female mouse sexual behavior
Source: PLoS One. 2024 Jun 20;19(6):e0304703. doi: 10.1371/journal.pone.0304703 (PMC11189176; doi:10.1371/journal.pone.0304703)
Supplement: S3 Fig — Survival analysis revealed significant differences, indicated by asterisks. *p < 0.05, **p < 0.01. (PDF) [file pone.0304703.s003.pdf]

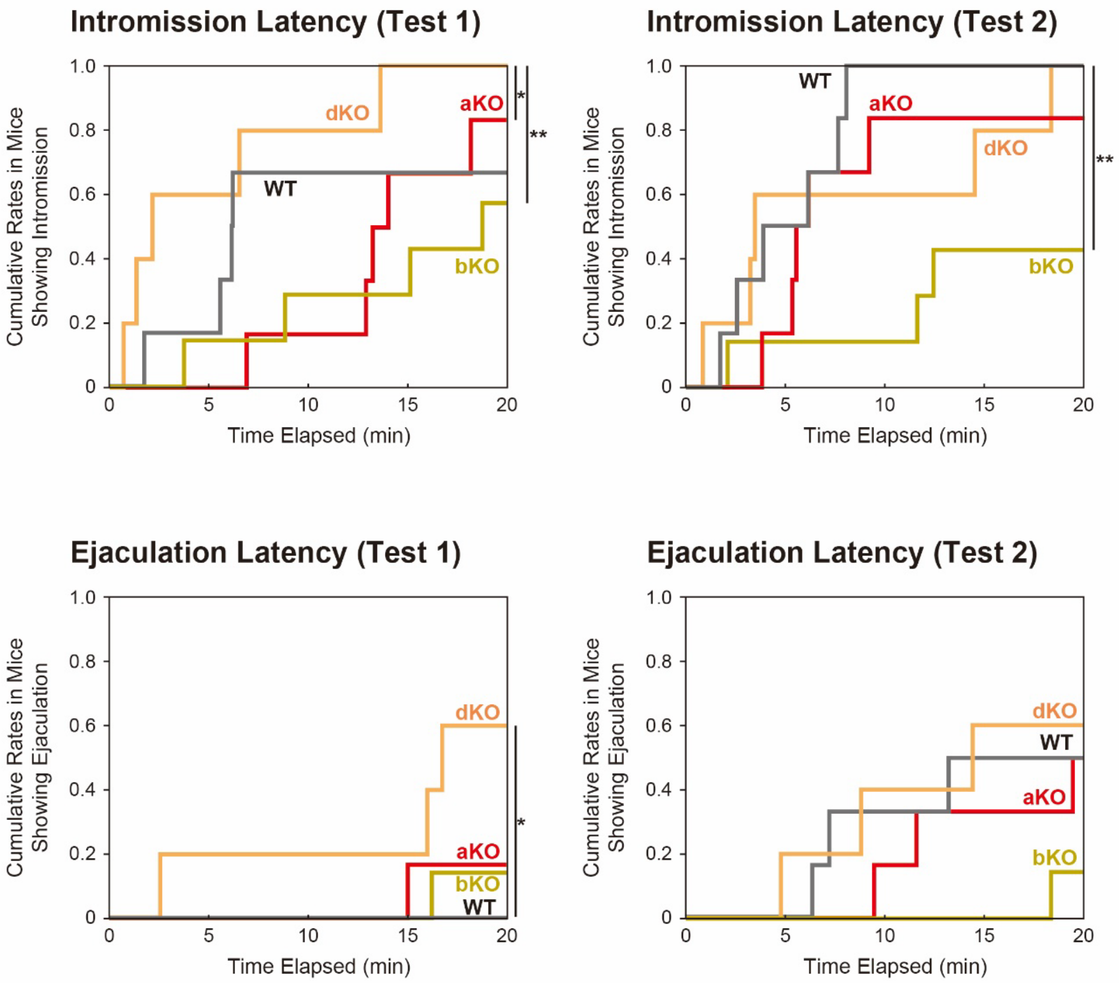
Intromission Latency (Test 1)
Intromission Latency (Test 2)
Ejaculation Latency (Test 1)
Ejaculation Latency (Test 2)
Cumulative Rates in Mice Showing Intromission
Cumulative Rates in Mice Showing Ejaculation
Time Elapsed (min)
0
0.2
0.4
0.6
0.8
1.0
0
5
10
15
20
dKO
aKO
WT
bKO
*
**
