# Supplementary material for: The bilevel chamber revealed differential involvement of vasopressin and oxytocin receptors in female mouse sexual behavior
Source: PLoS One. 2024 Jun 20;19(6):e0304703. doi: 10.1371/journal.pone.0304703 (PMC11189176; doi:10.1371/journal.pone.0304703)
Supplement: Attachment — Submitted filename: Review_Report_PONE-D-23-41361.pdf [file pone.0304703.s005.pdf]

In the research article entitled “The bilevel chamber revealed differential involvement of vasopressin and oxytocin receptors in female mouse sexual behavior”, the authors attempted to measure the female mice sexual behavior by constructing a new apparatus “bilevel chamber”. Also, they evaluated the effects of the arginine vasopressin (AVP) receptors v1a and v1b, and the oxytocin (OT) receptors on female mice sexual behavior in mice, by making the corresponding knockout female mice. Authors took the advantage of the easy genetic manipulation in mice to overcome the limitations (in comparison to rats) in studying the female mice sexual behavior. However, there are some concerns observed and the following minor and major issues listed.

Comments:

1) page 2:
a) line 10: The aKO females showed **decreased** rejection-like behavior, based on the discussion on page 20, lines 360-361, and Fig. 4A. Replace “increased” with “decreased”.

2) page 3:
a) line 22: Replace “evolutionary” with “evolutionarily”.
b) lines 31-33: Cite the references 9 and 10 after the word MPOA; or Cite all the references at the end of the sentence.

3) page 4:
a) line 40: The word “typical” is repeated twice. It may be corrected to one of the following:
“a typical diagnostic behavior” (OR) “a typical diagnostic type of behavior”.
b) line 47: Replace “Meanwhile” with “Also, it was reported/discovered that”.
c) line 51: It’s good to provide a figure for showing the sequence and structural similarity between AVP and OT. Otherwise give a reference.
d) lines 53-54: References should be given for lines 53 and 54.

4) page 5:
a) line 70: Use either “the” or “a”. Don’t use both, one after another.
b) line 74: The word “behavior” is repeated twice. This word maybe deleted at the end of the sentence.

5) page 6:
a) line 81: Give the “n” value.
b) lines 89-90: Give the brand/trade names for estradiol and progesterone used in this study.
c) line 89: Correct the “estradiol-17β benzoate” name appropriately to either “17β-estradiol 3-benzoate (EB)” or “17β-estradiol-3-benzoate (EB)”, in order to avoid confusion with the other estradiol benzoate variant “estradiol 17β-benzoate (E2-17B)”.
d) line 96: Replace “be adapt” with “get adapted”.

6) page 7:
a) line 98: Continue the sentence as “………..30 min, whichever is the first.”, if it’s appropriate.
b) lines 101-102: Give the LQ formula in a proper mathematical formula/equation format. Also, if you want to give this in the text, please express this correctly to “A lordosis quotient [LQ,

(number of lordosis / total number of mounts and intromissions received) × 100] was calculated…………”.
c) line 103: Modify the sentence to “………..repeated weekly until an LQ ≥ 50% was observed.”.
d) How many weeks did it take to observe an LQ ≥ 50%. It’s better if it’s mentioned here.
e) lines 110-112: How the constrained sideways, due to the narrow depth of the bilevel chamber, help the experimenter better evaluating the female lordosis. Is it by physically forcing/making the female mice body to squeeze as lordosis and/or rise its tail to better heights, due to space constraint. It’s good if it’s explained better.
Also, since it’s an external/instrumental physical/mechanical constraint on the mice body and its free movement, is it considered as disadvantageous for studying the true behavior of the mice. This constraint may act as a stimulus, similar to the male mount, and cause/create a false lordosis. I said false lordosis since it’s not because of the true stimulus (male mount). Is this a con/disadvantage of the newly designed apparatus (bilevel chamber).

7) page 8:
a) line 123: Use “the” after the word “Both”.
b) line 126: Replace “Then, when” with “While”, or with appropriate word.
c) line 128: Continue the sentence as “…………male ejaculation, whichever is the first.”, if it’s appropriate.
d) Why the ordinary cage test was carried out for 30 min (page 7, line 98) and the bilevel chamber test for 20 min (page 8, line 128).

8) page 9:
a) line 138: Remove “were obtained”, if it’s appropriate.
b) line 141: Use “the” after the word “classified”.
c) line 146: Is it possible for intromission event to happen without the lordosis event. How frequently this bout (Intromission without lordosis) was observed. Does this signifies that lordosis is not a necessary event to occur during mice mating/intercourse.
d) line 148: What is the minimum, maximum, average length of different bouts/events explained in this page. How could you measure the events with “> 2 sec” precision.
Also, how did you monitor and count/measure the no. of lordosis events in female mice; and the no. of mount, intromission, and ejaculation events in male mice. If it’s manual monitoring, how accurate is the counting.
e) lines 152-153: Give the IR formula in a proper mathematical formula/equation format. Refer the comment 6b (page 7).

9) page 10:
a) line 156: If the statement “IR was considered equally to LQ” was from the previously published literature, cite the suitable reference for this. And if this is your own observation/statement, explain why you considered this.
b) line 157: Correct appropriately to “……..LQ, as clearly distinguished…….”, if needed.
c) lines 161-163: Why the female lordosis events, and so the true LQ, could not be observed/measured accurately in the ordinary cage. How differently the bilevel chamber help the experimenter to observe the female lordosis events accurately. Just to mention here, the male mount, intromission, and ejaculation events are observed equally well in both the ordinary cage and bilevel chamber apparatus.

And, if the true LQ cannot be measured in the ordinary cage test, why do you consider the "estimated LQ", measured based on the IR and %Intro, to compare with the true LQ and %Intro calculated in the bilevel chamber test. Why don't you use the "%Intro" in place of "estimated LQ".
d) lines 168-169: Correct the sentence appropriately to "………and intromissions separately revealed that females exhibited…………".
e) line 172: Replace "behavior" appropriately with "behavioral", if needed.

10) page 11:
a) lines 180-181: Why the female rejection-like behavior events could not be observed/measured accurately in the ordinary cage. How differently the bilevel chamber help the experimenter to observe the female rejection-like behavior events accurately.
b) line 182: Remove the word "in".

11) page 12:
a) line 210: Use "and" before the word "dKO".
b) line 210: Why is the "n" value difference between the WT, aKO, bKO, and dKO mice groups.

12) page 13:
a) line 223: Does this line means that different stimulus males were used in the subsequent/different bilevel chamber tests. Make it clear.

13) page 14:
a) lines 244-245: The LQ formula/equation on page 14, lines 244-245 is different from that on page 7, lines 101-102. Correct this appropriately.
b) line 245: Replace "s" with "sec", for seconds.
c) Correct the following:
line 245: Mean duration
line 248: Total duration
Fig. 3B: Total duration

14) page 15:
a) lines 251-253: Explain it better. For example, in the test 1 also the lordosis duration was suppressed in bKO females compared to WT females.
b) line 259: Correct appropriately to "…post hoc analyses by Bonferroni test showed…….".
c) line 269: Figure title should be given in the Fig. 4 legend.

15) page 16:
a) Correct the following:
line 270: Mean % time
line 275: Time
Fig. 4B: % time
b) Lines 280, 283: Why is the "n" value difference between the WT, OTRKO, aKO, bKO, and dKO mice groups.

16) page 17:

a) line 296: Replace “s” with “sec”, for seconds.
b) line 304: How do you explain the effect of test replication.

17) page 18:
a) line 308: Figure title should be given in the Fig. 6 legend.
b) Correct the following:
line 309: Mean % time
line 314: Time
Fig. 6B: % time
c) What is the significance of % time spent with males, in the context of female sexual behavior.

18) page 19:
a) line 328: If the v1b receptor is not essential for lordosis expression, then why bKO showed almost no lordosis. Interpret this in a better way.
b) line 330: “in dKO females, aKO restored lordosis from the suppression by bKO up to the WT level”. How do you justify this hypothesis. Any experimental evidence. And how the dKO showed normal levels of lordosis, while the bKO showed no lordosis. It means there may be elements (other than v1a and v1b receptors) involving in nullifying/inhibiting the effect of bKO in presence of aKO. Can you shed light on this. If you propose that the crosstalk between OT and v1b receptors (page 20, line 356) maybe the underlying cause for this, please explain it better. And if so, how did you propose this hypothesis, and any attempts made for experimental evidence. Any other elements may involve in this ?.

19) page 21:
a) How the OTRKO females exhibit normal lordosis levels, while showing increased rejection-like behavior. What is the stimulus for lordosis in this case, if it’s not the male mount (which is not possible due to rejection by female mice).
b) lines 378-379: Does the “rejection-like behavior” reflect “proceptivity”. Please explain this.

20) Have you attempted to evaluate the effects of AVP, OT, AVP/OT combination, progesterone administrations/treatments in WT, aKO, bKO, dKO, OTRKO mice, similar to the rat lordosis experiments (page 19, lines 333-334; page 20, lines 352-353; page 21, lines 371).

21) Did you monitor and measure the Intromission and ejaculation events in experiments II and III.

22) Figure 2:
a) Replace “Ordinary” with “Ordinary Cage Test”, if appropriate
b) Replace “Bilevel Chamber” with “Bilevel Chamber Test”, if appropriate

23) P values and the corresponding bars should be given in the following figures.
a) Figure 3: Fig. 3B panel: P values and the corresponding bars for all the groups in Test 1.
b) Figure 4: Fig. 4B panel: P values and the corresponding bars for all the groups in Test 1 and 2.
c) Figure 5: Fig. 5A panel: P values and the corresponding bars for all the groups in Test 1 and 2.
