# Supplementary material for: The bilevel chamber revealed differential involvement of vasopressin and oxytocin receptors in female mouse sexual behavior
Source: PLoS One. 2024 Jun 20;19(6):e0304703. doi: 10.1371/journal.pone.0304703 (PMC11189176; doi:10.1371/journal.pone.0304703)
Supplement: Attachment — Submitted filename: PONE_D2341361R1_Review1_Report.pdf [file pone.0304703.s007.pdf]

In the revised version of the manuscript entitled “The bilevel chamber revealed differential involvement of vasopressin and oxytocin receptors in female mouse sexual behavior”, the authors made several edits as per the reviewers’ comments. I appreciate that the authors addressed all the issues raised by the reviewers, made all the suggested/necessary corrections throughout the text and figures, which improved the manuscript a lot. I think that the newly included extended discussions at multiple places improved the general understandability of the manuscript. These insertions also shed light on the future prospective and directions of the research in mice sexual behavior, regulatory mechanism(s), and the AVP & OT roles. I hope the “Bilevel chamber” devised in this research will be a useful tool in the future research. And I believe in my understanding that the revised manuscript maybe in good shape for publication with the following few minor modifications.

Minor Comments:

Line 56: Reframe the sentence to correct “with of nine”.
Line 95: Remove the extra space before the word “two”.
General comment: Please correct the page numbers in the responses to the reviewers’ comments, if the author responses along with the reviewer comments will be published as a separate section in the published manuscript.
