# Supplementary material for: The bilevel chamber revealed differential involvement of vasopressin and oxytocin receptors in female mouse sexual behavior
Source: PLoS One. 2024 Jun 20;19(6):e0304703. doi: 10.1371/journal.pone.0304703 (PMC11189176; doi:10.1371/journal.pone.0304703)
Supplement: Attachment — Submitted filename: Responses to REVIEWERs Comments.docx [file pone.0304703.s006.docx]

To Reviewer 1:

Thank you sincerely for your thoughtful and comprehensive review. We have thoroughly examined your comments and made revision to our MS accordingly. We believe that, thanks to your valuable advice, our MS has greatly improved. We trust that our corrections meet your criterion.

Below, we describe the changes we made to our MS following your comments. (an attached file contains changed points highlighted, each indicated your comment No.)

**1) page 2:**

**a)** line 10: The aKO females showed **decreased** rejection-like behavior, based on the discussion

on page 20, lines 360-361, and Fig. 4A. Replace “increased” with “decreased”.

Line 10: replaced it.

**2) page 3:**

**a)** line 22: Replace “evolutionary” with “evolutionarily”.

Line 10: replaced it.

b) lines 31-33: Cite the references 9 and 10 after the word MPOA; or Cite all the references at the

end of the sentence.

Line 32: The citation point was moved.

**3) page 4:**

**a)** line 40: The word “typical” is repeated twice. It may be corrected to one of the following:

“a typical diagnostic behavior” (OR) “a typical diagnostic type of behavior”.

Line 40: Thank you for pointing out our mistake. removed one.

**b)** line 47: Replace “Meanwhile” with “Also, it was reported/discovered that”.

Line 52: replaced it.

**c)** line 51: It’s good to provide a figure for showing the sequence and structural similarity between

AVP and OT. Otherwise give a reference.

Thank you for your advice. However, so ref#20 we cited contains a figure describing the sequences, that we did not think a similar figure is necessary in our MS.

d) lines 53-54: References should be given for lines 53 and 54.

Line 59: added citations.

**4) page 5:**

**a)** line 70: Use either “the” or “a”. Don’t use both, one after another.

Line 71: replaced it.

**b)** line 74: The word “behavior” is repeated twice. This word maybe deleted at the end of the

sentence.

Line 81: removed it.

**5) page 6:**

**a)** line 81: Give the “n” value.

Line 87: added “n” value.

**b)** lines 89-90: Give the brand/trade names for estradiol and progesterone used in this study.

Lines 96-97: added the brand name.

**c)** line 89: Correct the “estradiol-17β benzoate” name appropriately to either “17β-estradiol 3-

benzoate (EB)” or “17β-estradiol-3-benzoate (EB)”, in order to avoid confusion with the other

estradiol benzoate variant “estradiol 17β-benzoate (E2-17B)”.

Line 96: corrected it.

**d)** line 96: Replace “be adapt” with “get adapted”.

Line 103: changed it.

**6) page 7:**

**a)** line 98: Continue the sentence as “…………30 min, whichever is the first.”, if it’s appropriate.

Line 105: added your clause.

**b)** lines 101-102: Give the LQ formula in a proper mathematical formula/equation format. Also,

if you want to give this in the text, please express this correctly to “A lordosis quotient [LQ,

(number of lordosis / total number of mounts and intromissions received) × 100] was

calculated………….”.

Line 108: rewrote the equation.

**c)** line 103: Modify the sentence to “………..repeated weekly until an LQ ≥ 50% was observed.”.

Line 110: modified it following you.

**d)** How many weeks did it take to observe an LQ ≥ 50%. It’s better if it’s mentioned here.

Line 111: added the information.

**e)** lines 110-112: How the constrained sideways, due to the narrow depth of the bilevel chamber,

help the experimenter better evaluating the female lordosis. Is it by physically forcing/making the

female mice body to squeeze as lordosis and/or rise its tail to better heights, due to space constraint.

It’s good if it’s explained better.

Also, since it’s an external/instrumental physical/mechanical constraint on the mice body and its

free movement, is it considered as disadvantageous for studying the true behavior of the mice. This

constraint may act as a stimulus, similar to the male mount, and cause/create a false lordosis. I said

false lordosis since it’s not because of the true stimulus (male mount). Is this a con/disadvantage

of the newly designed apparatus (bilevel chamber).

Lines 118-121: we modified and added the description of the bilevel chamber. The depth of the apparatus constrained the body direction but did not prevent any behavior they showed.

**7) page 8:**

**a)** line 123: Use “the” after the word “Both”.

Line 136: inserted it.

**b)** line 126: Replace “Then, when” with “While”, or with appropriate word.

Line 139: replaced “Then, when" with "Subsequently,”

**c)** line 128: Continue the sentence as “…………male ejaculation, whichever is the first.”, if it’s

appropriate.

Line 141: added your clause.

**d)** Why the ordinary cage test was carried out for 30 min (page 7, line 98) and the bilevel chamber

test for 20 min (page 8, line 128).

In the initial phase of this study, we conducted sexual behavior tests for 30 min in the bilevel chamber. However, through several pilot tests, we determined that a 20-min test is sufficient to measure sexual behavior. Consequently, we have settled on this duration thereafter.

**8) page 9:**

**a)** line 138: Remove “were obtained”, if it’s appropriate.

Line 154: changed it.

**b)** line 141: Use “the” after the word “classified”.

Line 157: added “the”.

**c)** line 146: Is it possible for intromission event to happen without the lordosis event. How

frequently this bout (Intromission without lordosis) was observed. Does this signifies that lordosis

is not a necessary event to occur during mice mating/intercourse.

Lines 187-189: these are described in the Result.

**d)** line 148: What is the minimum, maximum, average length of different bouts/events explained

in this page. How could you measure the events with “> 2 sec” precision.

Also, how did you monitor and count/measure the no. of lordosis events in female mice; and the

no. of mount, intromission, and ejaculation events in male mice. If it’s manual monitoring, how

accurate is the counting.

Lines 170-172: In behavioral test, we have been using an event-recorder software (not commercial provided) to automatically measure the time and duration of events. The description of them was added in the Method. We did not calculate the average length in bouts (sorry), although raw data were stored in the software system. Calculating them requires some modification to the event-recorder programming.

**e)** lines 152-153: Give the IR formula in a proper mathematical formula/equation format. Refer

the comment 6b (page 7).

Lines 168-169: rewrote the equation.

**9) page 10:**

**a)** line 156: If the statement “IR was considered equally to LQ” was from the previously published

literature, cite the suitable reference for this. And if this is your own observation/statement, explain

why you considered this.

Lines 175-176: It is based on private communication with several researchers. Then, we modified the expressions of it. One example was cited.

**b)** line 157: Correct appropriately to “……..LQ, as clearly distinguished…….”, if needed.

Lines 175-176: corrected it.

**c)** lines 161-163: Why the female lordosis events, and so the true LQ, could not be

observed/measured accurately in the ordinary cage. How differently the bilevel chamber help the

experimenter to observe the female lordosis events accurately. Just to mention here, the male

mount, intromission, and ejaculation events are observed equally well in both the ordinary cage

and bilevel chamber apparatus.

And, if the true LQ cannot be measured in the ordinary cage test, why do you consider the

“estimated LQ”, measured based on the IR and %Intro, to compare with the true LQ and %Intro

calculated in the bilevel chamber test. Why don’t you use the “%Intro” in place of “estimated LQ”.

The sexual behavior of male mice has a characteristic movement pattern and is easily distinguished through visual observation. Conversely, assessing the presence or absence of female lordosis demands subtle judgment, considering factors such as tail position. In the ordinary cages, the direction of mouse bodies varied, making it challenging to consistently observe the angle of their tails.

**d)** lines 168-169: Correct the sentence appropriately to “……….and intromissions separately

revealed that females exhibited………….”.

Line 189: corrected it.

**e)** line 172: Replace “behavior” appropriately with “behavioral”, if needed.

Line 192: “behavior” was removed.

**10) page 11:**

**a)** lines 180-181: Why the female rejection-like behavior events could not be observed/measured

accurately in the ordinary cage. How differently the bilevel chamber help the experimenter to

observe the female rejection-like behavior events accurately.

Lines 121-125: We added the explanation why the female rejection-like behavior is hard to be observed in the ordinary cage in the Method.

**b)** line 182: Remove the word “in”.

Line 202: the sentence is modified.

**11) page 12:**

**a)** line 210: Use “and” before the word “dKO”.

Line 229: added “and”

**b)** line 210: Why is the “n” value difference between the WT, aKO, bKO, and dKO mice groups.

As demonstrating in this study, mice deficient in genes of AVP or OT receptors often faced challenges in reproduction, making it difficult to constantly obtain a specific number of mice. Consequently, we conducted behavioral tests simultaneously in subsets of subjects consisting of all experimental groups, and repeated the process. The variability in the number of mice among groups is a result of these circumstances.

**12) page 13:**

**a)** line 223: Does this line means that different stimulus males were used in the

subsequent/different bilevel chamber tests. Make it clear.

Lines 242-243: rewrote the sentence.

**13) page 14:**

**a)** lines 244-245: The LQ formula/equation on page 14, lines 244-245 is different from that on

page 7, lines 101-102. Correct this appropriately.

Lines 264-265: corrected it.

**b)** line 245: Replace “s” with “sec”, for seconds.

Lins 266: corrected it.

**c)** Correct the following:

line 245: Mean duration

Line 265: corrected it.

line 248: Total duration

Fig. 3B: Total duration

**14) page 15:**

**a)** lines 251-253: Explain it better. For example, in the test 1 also the lordosis duration was

suppressed in bKO females compared to WT females.

Lines 273-274: added a sentence to mentioned it.

**b)** line 259: Correct appropriately to “…post hoc analyses by Bonferroni test showed…….”.

Line 281: corrected it.

**c)** line 269: Figure title should be given in the Fig. 4 legend.

Lines 291-292: gave it a title.

**15) page 16:**

**a)** Correct the following:

line 270: Mean % time	line 294: done.

line 275: Time	line 298: done.

Fig. 4B: % time	Fig. 4B: done.

**b)** Lines 280, 283: Why is the “n” value difference between the WT, OTRKO, aKO, bKO, and

dKO mice groups.

The same issue of Comment#11b

**16) page 17:**

**a)** line 296: Replace “s” with “sec”, for seconds.

Line 319: replaced it.

**b)** line 304: How do you explain the effect of test replication.

Lines 391-394: added the explanation about the effect of experience on rejection-like behavior.

**17) page 18:**

**a)** line 308: Figure title should be given in the Fig. 6 legend.

Lines 331-332: gave it a title.

**b)** Correct the following:

line 309: Mean % time	line 334: done

line 314: Time	line 338: done

Fig. 6B: % time	Fig. 6B: done

**c)** What is the significance of % time spent with males, in the context of female sexual behavior.

As discussed in lines 398-402, during the apparatus design phase, we expected that % time spent sharing the same floor with males would be reflect of pacing behavior in female mice. However, upon carefully observation of OTRKO females during tests, which had an impact on this, we concluded that the elevated % time spent with males in OTRKO females resulted from prolonged interaction (rejection-like behavior) rather than changes in pacing behavior.

**18) page 19:**

**a)** line 328: If the v1b receptor is not essential for lordosis expression, then why bKO showed

almost no lordosis. Interpret this in a better way.

**b)** line 330: “in dKO females, aKO restored lordosis from the suppression by bKO up to the WT

level”. How do you justify this hypothesis. Any experimental evidence. And how the dKO showed

normal levels of lordosis, while the bKO showed no lordosis. It means there may be elements

(other than v1a and v1b receptors) involving in nullifying/inhibiting the effect of bKO in presence

of aKO. Can you shed light on this. If you propose that the crosstalk between OT and v1b receptors

(page 20, line 356) maybe the underlying cause for this, please explain it better. And if so, how

did you propose this hypothesis, and any attempts made for experimental evidence. Any other

elements may involve in this ?.

Lines 354-359: In response to your Comments 18a and 18b, we have further elaborated on our speculation in Discussion.

**19) page 21:**

**a)** How the OTRKO females exhibit normal lordosis levels, while showing increased rejection-like

behavior. What is the stimulus for lordosis in this case, if it’s not the male mount (which is

not possible due to rejection by female mice).

Lines 380-381: added a sentence about our explanation.

**b)** lines 378-379: Does the “rejection-like behavior” reflect “proceptivity”. Please explain this.

Lines 416-417: modified expression. The reduction of rejection-like behavior reflect “proceptivity”.

**20)** Have you attempted to evaluate the effects of AVP, OT, AVP/OT combination, progesterone

administrations/treatments in WT, aKO, bKO, dKO, OTRKO mice, similar to the rat lordosis

experiments (page 19, lines 333-334; page 20, lines 352-353; page 21, lines 371).

Lines 409-412: We added further prospects in study investigating the role of AVP and OT receptors on female sexual behavior.

**21)** Did you monitor and measure the Intromission and ejaculation events in experiments II and III.

Yes. We can add the data of male sexual behavior as the supplement.

**22)** Figure 2:

a) Replace “Ordinary” with “Ordinary Cage Test”, if appropriate

Replaced it.

b) Replace “Bilevel Chamber” with “Bilevel Chamber Test”, if appropriate

Replaced it.

**23)** P values and the corresponding bars should be given in the following figures.

a) Figure 3: Fig. 3B panel: P values and the corresponding bars for all the groups in Test 1.

P values appear in the Fig. legend.

b) Figure 4: Fig. 4B panel: P values and the corresponding bars for all the groups in Test 1 and 2.

P values appear in the Fig. legend.

c) Figure 5: Fig. 5A panel: P values and the corresponding bars for all the groups in Test 1 and 2.

P values appear in the Fig. legend.

To Reviewer 2:

Thank you very much for your evaluation. We appreciate your valuable advice immensely. Also, pointing out our mistakes has been incredibly helpful. We have revised the manuscript based on your comments, so please review it. The changes are highlighted, and each comment is numbered for your reference.

1) Figure 3A and b WT upper significance bar misleading, it looks like WT mice have statistical differences with both aKO and bKO, while the difference is only with bKO. Please check and modify the significance bars.

Thank you very much for pointing out our mistakes. These were corrected.

2) While the results of rejection-like behavior are clear, the influence of V1A and V1B receptors on lordosis seems puzzling. May you address more in the discussion why the absence of both receptors has the same results as their presence? While it seems to be some kind of interaction, it’s still unclear how knockout of V1AR can recover the effects of knockout of V1BR.

Lines 355-367: We included a description of our hypothesis deduced from the results.

3) Why you choose a different strain for experiment I? (C57BL6 for II and III is clear to me, because KO mice are based on the same strain)

Immediately after developing the bilevel chamber, we conducted a sexual behavior test using ICR female mice, known for their high sexual activity based on our experience, to verify its effectiveness. When preparing the paper, we recognized the importance of providing a thorough description of our new test equipment. Consequently, we decided to present the data as Experiment I, despite using a different mouse strain.

4) You used the terms receptivity and proceptivity in the conclusion and briefly used them in the discussion; May you clearly state in the introduction what you mean by those terms?

Lines 42-47: Thank you for your valuable advice. We delved more deeply into the concepts of "receptivity" and "proceptivity" in the Introduction.

5) May you indicate in your methods part how you clean the Believel chamber at the end of the test in order to remove odor influence between tests?

Lines 142-145: added a description of our cleaning process following each test trial.
