# Supplementary material for: The bilevel chamber revealed differential involvement of vasopressin and oxytocin receptors in female mouse sexual behavior
Source: PLoS One. 2024 Jun 20;19(6):e0304703. doi: 10.1371/journal.pone.0304703 (PMC11189176; doi:10.1371/journal.pone.0304703)
Supplement: Attachment — Submitted filename: Responses to REVIEWERs Comments 2.docx [file pone.0304703.s008.docx]

To Reviewer 1:

Thank you sincerely for your kind and detailed review. We made the correction following your comments.

We believe that our corrections meet your criterion.

Reviewer’s comments

Line 56: Reframe the sentence to correct “with of nine”.

Thank you. We rewrote the sentence.

Line 95: Remove the extra space before the word “two”.

Thanks again. We removed the extra one.
